# Small extracellular vesicle PD-L1 in cancer: the knowns and unknowns

**DOI:** 10.1038/s41698-022-00287-3

**Published:** 2022-06-21

**Authors:** Zi-Li Yu, Jin-Yuan Liu, Gang Chen

**Affiliations:** 1 The State Key Laboratory Breeding Base of Basic Science of Stomatology (Hubei-MOST) & Key Laboratory of Oral Biomedicine Ministry of Education, School and Hospital of Stomatology, Wuhan University, Wuhan, 430079 China; 2 Department of Oral and Maxillofacial Surgery, School and Hospital of Stomatology, Wuhan University, Wuhan, 430079 China; 3 Frontier Science Center for Immunology and Metabolism, Wuhan University, Wuhan, 430071 China

**Keywords:** Tumour immunology, Cancer

## Abstract

According to the conventional wisdom, programmed death protein 1 ligand (PD-L1)-mediated immunosuppression was based on the physical contact between tumor cells and T cells in the tumor microenvironment. Recent studies demonstrated that PD-L1 was also highly expressed on the surface of tumor cell-derived small extracellular vesicles (sEVs). PD-L1 on sEVs, which could also directly bind to PD-1 on T cells, has a vital function in immunosuppression and immunotherapy resistance. Due to the heterogeneity and dynamic changes of PD-L1 expression on tumor cells, developing sEV PD-L1 as a predictive biomarker for the clinical responses to immunotherapy could be an attractive option. In this review, we summarized and discussed the latest researches and advancements on sEV PD-L1, including the biogenesis and secretion mechanisms, isolation and detection strategies, as well as the biological functions of sEV PD-L1. In the meantime, we highlighted the application potential of sEV PD-L1 as diagnostic and prognostic markers in tumor, especially for predicting the clinical responses to anti-PD-1/PD-L1 immunotherapies. In particular, with the gradual deepening of the studies, challenges and problems regarding the further understanding and application of sEV PD-L1 have begun to emerge. Based on the current research status, we summarized the potential challenges and possible solutions, and prospected several key directions for future studies of sEV PD-L1. Collectively, by highlighting the important knowns and unknowns of sEV PD-L1, our present review would help to light the way forward for the field of sEV PD-L1 and to avoid unnecessary blindness and detours.

## Introduction

Programmed cell death protein-ligand 1 (PD-L1) is an immune checkpoint molecule that interacts with programmed cell death protein-1 (PD-1) to negatively regulate the proliferation and activation of immune cells. Under normal physiological conditions, PD-L1-mediated immunosuppression maintains immune homeostasis, thereby protecting normal cells from unnecessary damage. While in tumors, the PD-L1/PD-1 signaling axis is one of the major immune escape mechanisms mediating tumor progression. According to the conventional wisdom, PD-L1-mediated immunosuppression was mostly based on the direct physical contact between tumor cells and tumor-infiltrating T cells.

Recently, we and other researchers have revealed that PD-L1 is enriched on the surface of tumor cell-derived small extracellular vesicles (sEVs)^1–3^, which are membrane structures with a diameter smaller than 200 nm. Moreover, sEV PD-L1 seemingly recapitulates the effect of cell surface PD-L1; it can also directly bind to PD-1 on T cells and has a vital function in immunosuppression and tumor progression. However, differences do exist between the sEV PD-L1 and cell surface PD-L1. First, the relative level of PD-L1 on sEVs was generally higher than that on their parental cells. Cancer cells can secret a vast majority of their PD-L1 on sEVs rather than express PD-L1 on their own surface^2^, which may partially explain the phenomenon that sEV PD-L1 was detected in all patients but only 67% of their tumor biopsies were positive for PD-L1 expression^4^. Second, cell surface PD-L1-mediated inhibition of immunocytes occurs mainly in tumor site, whereas sEVs carrying PD-L1 can be secreted into circulation in large quantities, inducing systemic immunosuppression in the whole body. Third, due to the small volume and large specific surface area of sEVs, sEV PD-L1 can enter deep tissues and play unique functions. Recently, circulating sEV PD-L1 in peripheral blood was expected to be a liquid biopsy biomarker for tumor diagnosis, especially for predicting the patient response to immunotherapy. In addition, sEV PD-L1 is emerging as an important target for tumor therapy. However, the detailed functions and mechanisms of sEV PD-L1 in tumor immune escape remain elusive. Also, how it may be used as biomarker for the management of tumor or to define the subset of patients who would benefit from the immunotherapy is still unclear. Moreover, as studies move along, challenges and problems in the research and application of sEV PD-L1 making their future directions even more confusing.

In this review, we, therefore, summarized the recent studies on sEV PD-L1, including current knowledge on the biogenesis and secretion mechanisms, isolation and detection strategies, as well as the biological functions of sEV PD-L1. We also discussed the application potential of sEV PD-L1 as diagnostic and prognostic markers in tumor, especially for predicting the clinical responses to immunotherapies. More importantly, we highlighted several future key directions for sEV PD-L1, with particular emphasis on (a) selective purification of PD-L1-positive sEVs with natural properties and functions, (b) heterogeneity of PD-L1-positive sEVs, (c) standardization of sEV PD-L1-based liquid biopsy, and (d) novel tumor therapies based on sEV PD-L1.

## Biogenesis, trafficking, and secretion mechanisms of sEV PD-L1

**Fig. 1:**
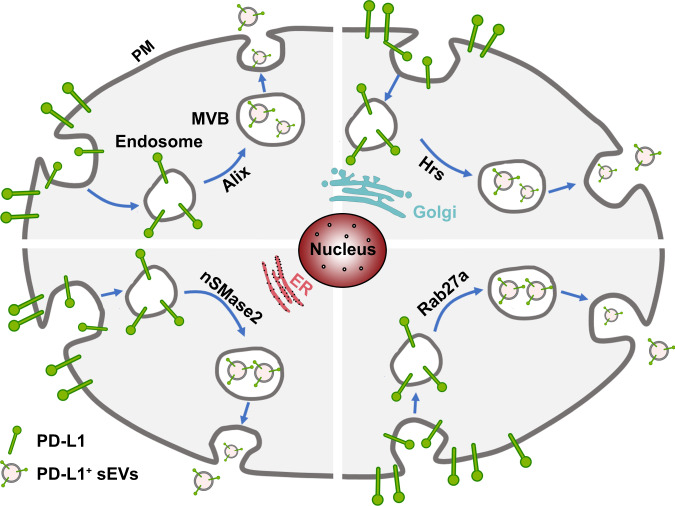
Biogenesis, trafficking, and secretion mechanisms of sEV PD-L1. PD-L1 was shifted from plasma membrane (PM). The early endosomes were formed by the internalization of the plasma membrane. Then, early endosomes undergo the process of mature. The early endosomal membrane buds inward to form small intraluminal vesicles (ILV), which also known as late endosome or multi-vesicular body (MVB). Finally, MVBs fuse with the plasma membrane to release ILVs containing PD-L1, which is known as sEV PD-L1.

In order to reveal the molecular mechanisms involved in the biogenesis, trafficking, and secretion of sEV PD-L1, following questions need to be answered. First, where is the sEV PD-L1 originated from? The study by Poggio et al. suggested that sEV PD-L1 may originate from the surface of donor cells, rather than directly from the endoplasmic reticulum (ER) or Golgi^2^. Monypenny et al. further revealed that the level of sEV PD-L1 was correlated with the level of membrane PD-L1 on donor cells, and Alix controlled the balance of PD-L1 distribution between cells and sEVs^5^. Knockdown of *ALIX* reduced sEV secretion of PD-L1 and correspondingly increased the level of cell surface PD-L1 by restricting the incorporation of PD-L1 into sEVs. These results indicate that plasma membrane PD-L1 of parent cells may be the major source of sEV PD-L1. As shown in Fig. 1, PD-L1 is shifted from plasma membrane to early endosomes, which are formed by the internalization of the plasma membrane. Then, early endosomes undergo the process of mature. The early endosomal membrane buds inward to form small intraluminal vesicles (ILVs), also known as late endosome or multi-vesicular body (MVB)^6^. Finally, MVBs fuse with the plasma membrane to release ILVs containing PD-L1, which is known as sEV PD-L1. Nevertheless, further researches, especially the composition analysis of PD-L1-positive sEVs, are needed to explore and confirm new subcellular origin of sEV PD-L1 beyond cell surface PD-L1.

Second, which are the key regulators involved in the biogenesis, trafficking, and secretion of sEV PD-L1? Endosomal sorting complex required for transport (ESCRT)-mediated membrane invagination and scission are critical steps in biogenesis of sEVs^7^. Protein cargos can be captured onto the membrane of endosomes and then sorted into ILVs by the ESCRT machinery. ESCRT-0, ESCRT-I, ESCRT-II, and ESCRT-III components as well as several accessory components act in a stepwise manner, where they respectively charge the ubiquitination, sorting, trafficking, and secretion of transmembrane cargos^6^. Our study demonstrated that PD-L1 colocalized and traveled with the ESCRT-0 subunit Hrs in MVBs, suggesting that Hrs mediated the recognition and sorting of PD-L1^1^. Moreover, Hrs co-immunoprecipitated with PD-L1 from whole-cell lysates^1^. Importantly, knockdown of *HGS* (gene of Hrs) significantly blocked the secretion of sEV PD-L1^1,8^, suggesting an essential role of Hrs in the biogenesis and secretion of sEV PD-L1. On the other hand, extensive studies have demonstrated that Rab family members play a vital role in the biogenesis, transporting, and secretion of sEVs^9,10^. Recent studies, including ours, revealed that the secretion of sEV PD-L1 was Rab27a-dependent. Knockdown of *RAB27A* obviously reduced the secretion of sEV PD-L1^1,2^. Additionally, nSMase2, a rate-limiting enzyme of ceramide biosynthesis, has been also implicated in the secretion of sEV PD-L1^2^. GW4869, a neutral sphingomyelinase inhibitor for nSMase2, could reduce sEV secretion as well as the level of PD-L1 on sEVs, while had no effect on PD-L1 expression in cells^8,11^. The results indicate that ESCRT components, Rab family members, and nSMase2 may be involved in the sorting and trafficking of PD-L1 into ILVs, and play critical roles in the biogenesis and secretion of sEV PD-L1. Based on the available data, however, it seems that the molecular mechanisms or signal pathways that regulate the biogenesis and secretion of sEV PD-L1 may vary across different cell types and even status of cells.

The secretion of sEV PD-L1 could be also enhanced by external stimulus. The level of sEV PD-L1 secreted by melanoma cells was increased markedly in response to IFN-γ treatment^1^. Of note, IFN-γ treatment did not increase the number of secreted vesicles^2^, suggesting that IFN-γ enhanced PD-L1 level of single sEV other than enhanced the secretion of sEVs. In addition, Wu et al. revealed that smoking could excite the secretion of sEVs and increased the level of sEV PD-L1 in bronchial lavage fluid derived from non-small cell lung cancer (NSCLC) patients^12^.

## Isolation and detection of sEV PD-L1

### Isolation of sEVs

**Fig. 2:**
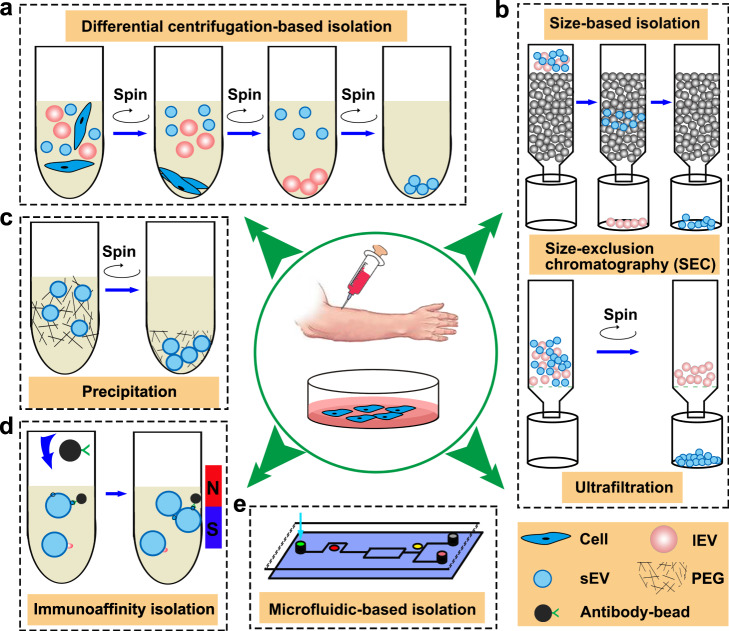
The isolation methods of sEVs. Isolation methods of sEVs were divided into differential centrifugation-based isolation (**a**), size-based isolation technique (**b**), precipitation (**c**), immunoaffinity isolation (**d**) and microfluidic-based isolation (**e**).

Several strategies have been developed for the isolation of sEVs with appreciable quantity and purity, including differential centrifugation (DC)-based isolation techniques, size-based isolation techniques, precipitation-based techniques, immunoaffinity capture-based isolation techniques, and microfluidics-based isolation techniques (Fig. 2). Each technique exploits a particular trait of sEVs, such as their density, shape, size, and surface proteins to aid their isolation. The principles, advantages, and disadvantages of the current isolation techniques for sEVs is summarized in Table 1.

**Table 1:** The current isolation techniques for sEVs.

Isolation technique	Isolation principle	Potential advantage	Potential disadvantage
Ultracentrifugation-based technique	Particulates in suspension will be sedimented according to their density, size, and shape when subjected to a centrifugal force	Easy to operate, need no special reagent, large sample capacity and yield large amounts of sEVs	Protein aggregates contamination, high shear force may induce the aggregation and rupture of sEVs, high equipment cost, instruments consume a great deal of space, long run time
Size-based technique	Based on the size difference between sEVs and other particulates in suspension	Ultrafiltration: need no special reagent, fast, low cost; SEC: harvest highly purified sEVs	Ultrafiltration: moderate purity, high shear force may induce rupture of sEVs, decrease yield when sEVs attached to filter; SEC: need special and customized equipment, time-consuming
Precipitation	Altering the solubility or dispersibility of sEVs with water-excluding polymers	Easy to operate, large sample capacity, need no special equipment	Protein aggregates contamination, take a long time to precipitation
Immunoaffinity capture-based technique	Specific binding between antigen tags of sEVs and immobilized antibodies	Simple and convenient strategy, harvest highly purified sEV subtyping, short run time	High reagent cost, only a portion of the sEVs can be separated (low yields), antigen tags were blocked by reagents, which affects the biological behaviors of the isolated sEVs
Microfluidic-based technique	Immunoaffinity, size, or density were integrated into the microfluidic chip	Microscale isolation and need little amount of body fluid samples (dozens of microliters), integrate separation and detection into a single chip, fast and easy automation	Low sample capacity, need special and customized regents, lack of standardization tests on clinical samples

*SEC* size exclusion chromatography.

As the current gold-standard method for sEV isolation, DC usually consists of a series of cycles of different centrifugal force and duration to isolate sEVs based on their density and size differences from other components in a sample (Fig. 2a). For ultracentrifugation, the centrifugal force typically ranges from 100,000 g to 120,000 g. Despite being widely used, DC has several drawbacks such as bulky and costly instrumentation as well as time-consuming procedures. Moreover, non-sEV contaminants (e.g., protein aggregates) that share similar physical properties with sEVs could not be eliminated using centrifugation. Most importantly, the yield of sEVs isolated by DC was typically low (5–40% of total sEVs)^11,13^. Two popular size-based sEVs isolation techniques are ultrafiltration and size exclusion chromatography (SEC) (Fig. 2b). In SEC, a porous stationary phase is used to sort sEVs out according to their size. Components with small hydrodynamic radius in sample can pass the pores, resulting in late elution. By altering their solubility or dispersibility, sEVs can be force out of solution. In precipitation-based isolation, water-excluding polymers such as polyethylene glycol (PEG) was used to tie up water molecules and force less soluble components out of the solution (Fig. 2c).

Interestingly, it was reported that the level of sEV PD-L1 was highly dependent on the isolation methods, for instance sEVs isolated by SEC carried more surface PD-L1 than sEVs isolated by DC^14^. It was also indicated that sEVs isolated by SEC may have improved biological function compared to their counterparts isolated by DC^15,16^. Moreover, circulating sEV PD-L1 isolated by DC was correlated with tumor volumes in glioblastoma patients^17^. However, the level of sEV PD-L1 isolated by serial density gradient ultracentrifugation (DGU) showed no significant difference between glioma patients and healthy donors^18^.

Studies have shown that sEVs in physiological fluids and tissues represent a heterogeneous mixture of sEVs with different surface markers which secreted by various parental cells. Purification of PD-L1-positve sEVs from heterogeneous sEVs is essential to explore their biological function. Heterogeneous sEVs, regardless of PD-L1 expression, were isolated by DC, size-based isolation, and precipitation-based isolation. Those methods were not ideal for the specific isolation and targeted study of PD-L1-positive sEVs. Instead, the immunoaffinity-based isolation, which is able to selectively capture of sEVs that bear indicated surface markers, is useful for specific isolation of PD-L1-positive sEVs (Fig. 2d). Thanks to the rapid progress in microfabrication technology, microfluidic-based isolation has been recently performed for the rapid and efficient isolation of sEVs on both the physical and biochemical properties of sEVs at microscales (Fig. 2e). Microfluidics-based devices could integrate the capture and detection of sEV PD-L1 into a single chip.

### Detection of sEV PD-L1

#### Conventional detection methods

**Fig. 3:**
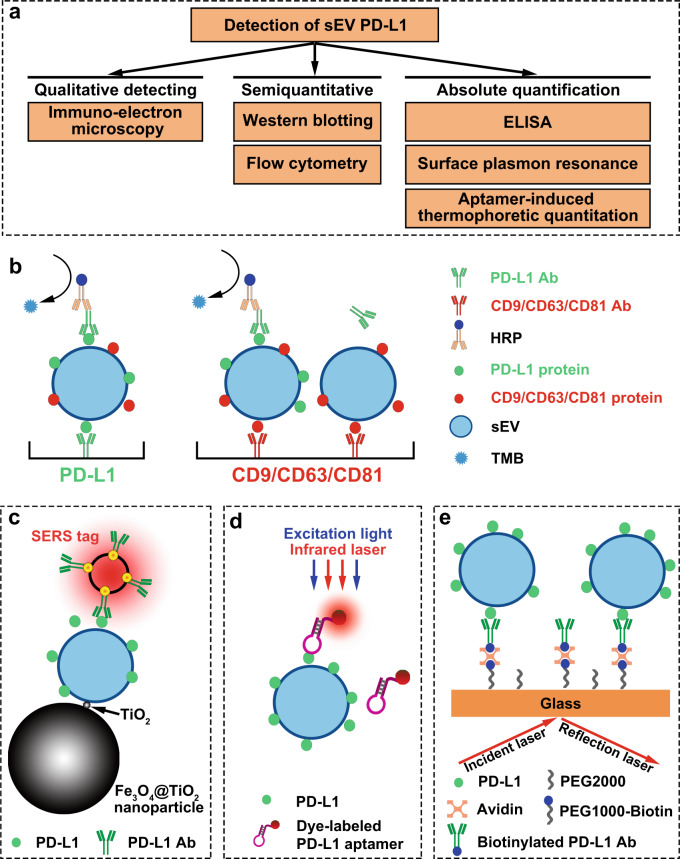
The detection strategies of sEV PD-L1. **a** The detection methods of sEV PD-L1 were divided into three categories based on the qualitative detection and quantitative determination. **b** Schematic of ELISA to measure PD-L1 levels on the surface of sEVs. **c** PD-L1 level were quantified by anti-PD-L1 antibody-modified Au@Ag@MBA surface-enhanced Raman scattering (SERS) tags. **d** An efficient and sensitive quantitation method for circulating sEV PD-L1 using aptamer-induced thermophoretic quantitation. **e** Surface plasmon resonance (SPR) biosensor to detect the sEV PD-L1.

The detection methods of sEV PD-L1 could be divided into three categories, namely the qualitative, semiquantitative, and absolute quantitative detection (Fig. 3a). Electron microscopy, which revealed cup-shaped morphologies characteristic of sEVs, is a qualitative detection method. Immuno-electron microscopy was also used to demonstrate that the extracellular domain of PD-L1 was exposed on the surface of sEVs. PD-L1 was anchored on the surface of some but not of all sEVs^17^ and the percentage of PD-L1-positive sEVs could be conveniently measured by nanoscale flow cytometry^18^. When magnetic beads or latex beads were used to capture sEVs, PD-L1 levels of sEVs/bead complexes can be measured with traditional flow cytometry^19,20^. Additionally, the relative quantification of sEV PD-L1 could also be presented by the relative fluorescence intensity of PD-L1-conjugated fluorescein. Western blotting was also a semiquantitative method used to evaluate the level of total PD-L1 protein in sEVs. While, sEVs were dissolved by lysis buffer and the spatial position of PD-L1 could not be detected by western blot analysis.

The absolute quantification of the level of sEV PD-L1 was usually performed by enzyme linked immunosorbent assay (ELISA). As shown in Fig. 3b, there are two ELISA strategies to detect sEV PD-L1. The key distinction between the two strategies is the capture antibody. The capture antibody of the two strategy is anti-PD-L1 antibody and anti-surface marker antibody, respectively. Western blotting and ELISA have detection limit of ng and pg respectively, which was hard to detect the very low abundance of sEV PD-L1 at the early stage of tumor^21^. Moreover, western blotting and ELISA assay required complex samples preparation time (>48 h) and relative large amount of body fluid samples to harvest sEVs.

#### New detection methods

The defects of current detection methods, such as time-consuming and low sensitivity have given rise to new detection methods of sEV PD-L1. Pang et al. reported a simple method integrating capture and analysis of sEV PD-L1 directly from serum of NSCLC patients^22^. Specifically, sEVs were enriched and separated from 4 μl serum sample by Fe_3_O_4_@TiO_2_ nanoparticles within 5 min with a capture efficiency of 96.5%. Then, the level of PD-L1 was quantified by anti-PD-L1 antibody-modified surface-enhanced Raman scattering (SERS) tags within 40 min with high sensitivity and low detection limit (one PD-L1-positive sEV/μl) (Fig. 3c). Huang et al. recently reported an efficient and sensitive method for detecting circulating sEV PD-L1 using aptamer-induced thermophoretic quantitation^23^ (Fig. 3d). Aptamer, with higher molecular recognition capability and higher binding efficiency to PD-L1 than antibody, can significantly improve the detection sensitivity of sEV PD-L1. They claimed that this method provided an accurate tumor diagnosis (AUC: 0.999) with the most appropriate cutoff value of 39 ng/ml. Its detection sensitivity was 11-times higher than that of ELISA. Furthermore, the total incubation and testing time took less than 1 min, which was far less than the overnight incubation and tedious washing time of the ELISA and western blot analysis.

Surface plasmon resonance (SPR) is a highly sensitive, label-free, and real-time optical detection method. Liu et al. demonstrated the feasibility of using compact SPR biosensor to detect sEV PD-L1 in the serum of NSCLC patients^24^ (Fig. 3e). They concluded that the compact SPR biosensor showed higher detection sensitivity than ELISA and similar sensing accuracy as ELISA. Additionally, microfluidic-based isolation techniques offer a promising way to integrate separation and detection of sEV PD-L1 into a single chip. Yang et al. developed an anti-PD-L1 antibody-tethered immune-biochip that could selectively capture PD-L1-positive sEVs from 30 μl serum of NSCLC patients^25^. The RNAs of interest in PD-L1-positive sEVs were quantified in the chip using cationic lipoplexes-containing molecular beacons within 4 h, showing superior performance in the diagnosis of lung cancer.

## The role of sEV PD-L1 in immunosuppression

### The immunosuppressive effects of sEV PD-L1 on T cells

#### sEV PD-L1 inhibited the activation of T cells

**Fig. 4:**
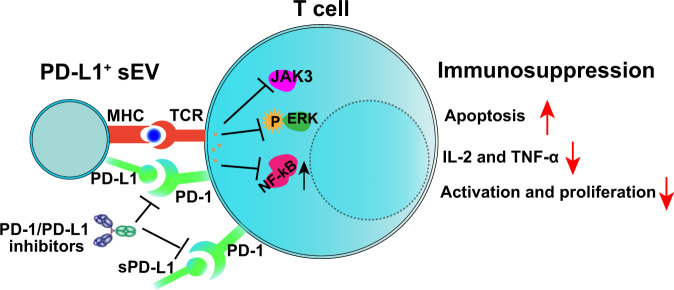
The immunosuppressive effects of sEV PD-L1. sEV PD-L1 acts as second signal and exerts inhibitor signals on T cells through PD-1, which promoted the apoptosis of T cells and suppressed the activation and production of cytokines.

PD-L1 acts as an important brake in anti-tumor immunity, which helps tumor cells evade immune destruction^26^. According to the conventional wisdom, PD-L1-mediated immunosuppression was based on the physical contact between tumor cells and T cells. Recently, researchers have demonstrated that PD-L1 was highly exogenously expressed on the surface of sEVs^1^. Importantly, sEV PD-L1 could directly bind PD-1 on the surface of both CD8 and CD4 T cells and inhibit T cell receptor (TCR)-mediated T cell activation (Fig. 4). Pre-treatment of sEVs with PD-L1 blocking antibodies nearly abolished the inhibiting effect of sEV PD-L1 on T cell activation^17^. Moreover, in vivo studies further revealed that sEV PD-L1 suppressed not only T cell activation in the tumor microenvironment and draining lymph node^1,2,19^, but also T cells at distance (e.g., spleen), or even in circulation.

#### sEV PD-L1 induced apoptosis and inhibited cytokine production of activated T cells

In addition to inhibiting the activation of T cells, Zhang and Kim evaluated the effects of sEV PD-L1 on activated T cells. They revealed that the apoptosis of activated T cells was significantly enhanced after exposure to PD-L1-positive sEVs compared with that of PD-L1-negative sEVs^27,28^. Importantly, the pro-apoptotic effect of sEV PD-L1 could be reversed by blockade of the PD-L1/PD-1 signaling^27,28^. Cytokines play critical roles in T cell-mediated anti-tumor immune. As expected, the cytokine (e.g., interleukin-2, INF-γ, and tumor necrosis factor) production of CD8 T cells was also inhibited by PD-L1-positive sEVs in a dose-dependent manner. This effect was restored by PD-L1 knockout or blocking PD-L1 on sEVs^1,3,28^. These results demonstrated that sEV PD-L1 can engage immune checkpoint pathways to inhibit cytokine production and induce apoptosis of T cells, supplementing the theory of PD-L1-mediated immunosuppression. Since sEV PD-L1-induced immunosuppression is usually accompanied by tumor progression, previous studies have claimed that sEV PD-L1 protects tumor cells and enhances tumor growth by inducing apoptosis and inhibiting cytokine production of activated T cells^3,28^.

#### The molecular mechanism of sEV PD-L1-mediated immunosuppression

The molecular mechanism by which sEV PD-L1 inhibits the function of T cells remains unclear. Previous studies have reported that immune-related signaling requires the “synapse” like structure. Indeed, tumor-derived sEVs have been shown to carry MHC molecules and adhesion molecules (e.g., ICAM-1), and integrins^29–31^. Moreover, MHC-I molecules on sEVs could promote T cell dysfunction and immunosuppression induced by sEV PD-L1^29^. This may explain the fact that the immunosuppressive effects of sEV PD-L1 were significantly stronger than those of soluble PD-L1 (sPD-L1), which did not carry MHC-I^29^. MHC molecules, which are essential ligands for TCR, induce the first activation signal of T cells. Then, PD-L1, which acts as second signal, exerts inhibitory signals after MHC interaction with TCR. However, how does the PD-L1-positive sEVs target and recognize T cells remains unclear, which needs to be further explored and improved. Of interest, a recent study has also reported that ICAM-1 and PD-L1 co-localize on sEVs and are both upregulated by INF-γ. More importantly, ICAM-1 is a prerequisite for sEV PD-L1-mediated inhibition of CD8 T cells^30^. Additionally, some other adhesion molecules such as CEACAM1 and ICAM-4 can also mediate the interaction between sEVs and CD8 T cells. These results indicate that the “synapse” like structure may be potentially formed between sEVs and T cells, while more convincing evidence is still needed.

According to the available reports, several signaling pathways may be involved in sEV PD-L1-mediated immunosuppression. Placenta-derived sEVs express PD-L1 and mediate T cell suppression by suppressing the CD3-zeta and Janus kinase 3 (JAK3) pathway, participating in the immune protection of the fetus^32^. Yang et al. revealed that PD-L1-positive sEVs but not PD-L1-negative sEVs significantly inhibited CD3/CD28-mediated ERK phosphorylation and NF-κB activation of T cells in a dose-dependent manner^3^. It would be interesting to evaluate how much sEVs are needed to achieve the T cell inhibition. Actually, the accurate molar ratio is rarely mentioned in previous reports. A recent study by Li and colleagues has reported that 10 μg PD-L1-positives EVs secreted by Wharton’s Jelly-derived mesenchymal stem cells (WJMSCs) efficiently inhibited TCR-mediated T cell activation in 1 × 10^5^ human PBMCs^33^. Consistently, we also previously reported that the functions of 2 × 10^5^ human CD8 T cells could be significantly inhibited by 25 μg human cancer cell-derived PD-L1-positive sEVs (carrying surface PD-L1 at a level of 0.05 ng per μg of sEVs)^1^. According to the limited available results, it seems that about 12 μg of sEVs carrying about 0.6 ng of surface PD-L1 are needed to achieve the inhibition of 1 × 10^5^ T cells in vitro. However, the exact amount of sEV PD-L1 needed to achieve T cell inhibition is still to be determined and verified by more studies. It can be speculated that the findings would be highly variable, because the immunosuppressive effects of sEV PD-L1 could be affected by several important factors, such as the cellular origin, the status of recipient cells, as well as the level of other proteins on sEVs, for instance, MHC-I and ICAM-1.

## sEV PD-L1 as a diagnostic and prognostic biomarker in cancer

It has been widely accepted that sEVs carry various functional molecules that are detected in their parental cells. Thus, the molecular profiles of sEVs can well recapitulate that of their parental cells. This is the theoretical basis of sEV-based liquid biopsy. Currently, the level of sEV PD-L1 as well as their clinical significance in different tumor types have been extensively investigated (Table 2). The reported results suggest that circulating sEV PD-L1 holds great promise to serve as a diagnostic and prognostic biomarker in cancer.

**Table 2:** sEV PD-L1 as a diagnostic and prognostic biomarker in cancer.

Cancer type	Sample type	Detection method of sEV PD-L1	Number of cases	Relationship between sEV PD-L1 and clinical features	Relationship between sEV PD-L1 and pathological features	Diagnostic and/or prognostic significance	References
Lung cancer	Serum	ELISA	85 (Stage I–IV)	The level of sEV PD-L1 in III/IV NSCLC patients was significantly higher than that of I/II NSCLC patients; the level of circulating sEV PD-L1 was positively correlated with tumor size	The level of circulating sEV PD-L1 was positively correlated with lymph node metastasis and distant metastasis	The feasibility of sEV PD-L1 to be used as a diagnostic biomarker for screening of early and late NSCLC patients needs to be further validated	^35^
		Compact surface plasmon resonance (SPR) biosensor and ELISA	5	The level of sEV PD-L1 in NSCLC patients was higher than that in HD	ND		^24^
		Surface-Enhanced Raman Scattering (SERS) immunoassay	17 (Stage I–IV)	The level of sEV PD-L1 in early (stage I-II) and late (stage III-IV) NSCLC patients was significantly higher than that in HD (*n* = 12)			^22^
		Immunobiochip	20 (Stage I–IV)	miR-21 and thyroid transcription factor-1 (TTF-1) mRNA in PD-L1-positive sEVs can distinguish HD from early and late NSCLC patients more sensitively and specifically than miR-21 and TTF-1 mRNA in whole serum			^25^
Head and neck squamous cell carcinoma	Plasma	Flow cytometry	40 (Stage I–IV)	The percentage and relative fluorescence value (RFVs) of PD-L1-positive sEVs in AD (*n* = 23) patients was significantly higher than that in NED (*n* = 17) patients; the percentage and RFVs of PD-L1-positive sEVs in late (stage III-IV) patients (*n* = 16) was significantly higher than that of early (stage I-II) patients (*n* = 24)	The percentage and RFVs of PD-L1-positive sEVs in patients with positive lymph nodes (*n* = 22) was significantly higher than that in patients with negative lymph nodes (*n* = 18)	sEV PD-L1 is potential to serve as biomarker for disease progression and clinical stage in HNSCC	^19^
		Flow cytometry	22 (Stage I–IV)	CD3^+^ sEV PD-L1 level, but not CD3^−^ fractions, from patients with late (stage III-IV) NSCLC was significantly elevated versus early (stage I-II) NSCLC patients	CD3^−^ sEV PD-L1 in patients with lymph node metastasis was significantly higher than patients with no lymph node metastasis		^38^
Gastric cancer	Serum	ELISA	69 (Stage I–III)	Higher baseline level of circulating sEV PD-L1 was associated with lower overall survival in 31 metastatic gastric cancer patients receiving chemotherapy; the level of sEV PD-L1 was an independent prognostic factor in gastric cancer patients	ND	sEV PD-L1 is a promising biomarker for predictive prognosis and immunosuppression status in metastatic gastric cancer patients	^29^
	Plasma	ELISA	21 (Stage III–IV)	Circulating sEV PD-L1 increased more significantly after 5-fluorouracil (5-FU) treatment in clinical non-responders compared with the responders			^27^
Pancreatic cancer	Serum	Flow cytometry	55	The median postoperative survival time of sEV PD-L1-positive pancreatic cancer (17.2 months) were significantly longer than sEV PD-L1-negative patents (7.84 months)	ND	sEV PD-L1 can serve as a negative prognostic factor for pancreatic cancer	^20^
Glioma	Plasma	Western blotting	19	PD-L1 was highly expressed in circulating sEVs from both stage IV glioblastoma patients and HD, and the difference was not significant	ND	sEV PD-L1 is not a reliable predictor in glioblastoma diagnosis	^18^
	Serum and plasma	Droplet PCR	21	The DNA level of sEV PD-L1 was positively correlated with the tumor volume (up to 60 mm^3^) and the level of PD-L1 in tumor tissue determined by IHC		Gene expression level of sEV PD-L1 is a potential marker in early diagnose of glioblastoma cancer	^17^
	Plasma	qRT-PCR	34	Glioma patients’ response to vaccination therapy can be predictive by sEV PD-L1 mRNA level			^46^
Other	Plasma	HOLMES-ExoPD-L1/ELISA	34 (15 Stage I–IV, urothelial carcinoma, 11 gastric adenocarcinoma, 6 prostate adenocarcinoma, 1 ovarian sarcoma, 1 SCLC)	The level of circulating sEV PD-L1 can effectively distinguish cancer patients from HD	sEV PD-L1 concentration was highly positively correlated with the metastasis	sEV PD-L1 is expected to be a predictor for disease progression in cancer	^23^

*AD* active disease, *NED* no evidence of disease, *HD* healthy donors, *ND* not defined, *SCLC* small cell lung cancer, *NSCLC* non-small cell lung cancer, *HNSCC* head and neck squamous cell carcinoma.

### Lung cancer

Worldwide, lung cancer remains the leading cause of cancer incidence and mortality^34^. Li et al. investigated the level of sEV PD-L1 and sPD-L1 in circulation as well as the expression level of PD-L1 in matched tissues in 85 patients with early-stage NSCLC^35^. They found that the levels of sEV PD-L1 were significantly different between NSCLC patients and healthy donors. Although the sEV PD-L1 level showed a slight correlation with sPD-L1 level, there was no difference in sPD-L1 level between NSCLC patients and healthy donors. In addition, the level of sPD-L1 was not correlated with any clinicopathologic features except for tumor size. However, the level of sEV PD-L1 of III/IV NSCLC patients were significantly higher than that of I/II NSCLC patients. Also, the level of sEV PD-L1 was positively correlated with tumor size, lymph node metastasis, and distant metastasis. Neither sEV PD-L1 nor sPD-L1 was associated with PD-L1 (clone of the used anti-PD-L1 antibody for IHC: 28-8) IHC profiles in tissues. Liu et al. also demonstrated the higher level of sEV PD-L1 in NSCLC patients than healthy donors^24^. Pang et al. revealed that the level of sEV PD-L1 in early (stage I–II) and late (stage III–IV) NSCLC patients were significantly higher than that of healthy donors (*n* = 12). Nevertheless, the difference was not significant between stage I–II (*n* = 7) and stage III–IV (*n* = 10) NSCLC patients^22^. Yang et al. investigated the level of miR-21 and thyroid transcription factor-1 (TTF-1) mRNA in PD-L1-positive sEVs from NSCLC patients. The receiver operating characteristic (ROC) curve showed that miR-21 and TTF-1 mRNA in PD-L1-positive sEVs showed higher sensitivity and specificity in distinguishing healthy donors from early stage and late-stage NSCLC patients than whole serum miR-21 and TTF-1 mRNA respectively^25^. These results suggest that PD-L1-positive sEVs have great potential to serve as biomarkers in lung cancer^36^.

### Head and neck squamous cell carcinoma (HNSCC)

HNSCC originates from the mucosal epithelium in the oral cavity, pharynx, and larynx and is one of the most common malignancies that arise in the head and neck region^37^. Theodoraki et al. isolated sEVs from the plasma samples of 40 HNSCC patients received surgery, radiotherapy, or chemotherapy^19^. They found that the level of sEV PD-L1, but not the level of sPD-L1, correlated with clinicopathologic parameters, including disease stage and lymph node status. Specifically, the percentages and relative fluorescence values (RFVs) of PD-L1-positive sEVs in patients with active disease (AD, *n* = 23) were significantly higher than that of no evidence of disease (NED, *n* = 17) patients. Moreover, the patients with positive lymph nodes (*n* = 22) or stage III-IV (*n* = 16) had significantly higher percentages or RFVs of PD-L1-positive sEVs than that of patients with negative lymph nodes (*n* = 18) and stage I–II (*n* = 24) respectively. In another study, the investigators separated T cell-derived sEVs from plasma of HNSCC patients using anti-CD3 antibody-conjugated magnetic beads^38^. Interestingly, the difference of percentage or relative fluorescence intensity of PD-L1-positive sEVs between the CD3^+^ and CD3^−^ fractions was not significant, indicating that T cell-derived sEVs carried similar level of PD-L1 compared with that of tumor cells. The sEV PD-L1 level in CD3^+^ sEVs, but not CD3^−^ sEVs, from patients with stage III-IV NSCLC was significantly higher than that of patients with stage I-II NSCLC. They also found the significant higher level of PD-L1 in CD3^−^ sEVs in patients with lymph node metastasis than those with no lymph node metastasis. The difference was, however, not significant for the level of PD-L1 in CD3^+^ sEVs between patients with or without lymph node metastasis. These results indicated that PD-L1-positive sEVs, whether produced by tumor cells or immune cells, have the potential to serve as a biomarker for disease activity and stage in HNSCC. However, it should be noticed that the aforesaid results were obtained with “on-bead flow cytometry”, where sEVs were bound to CD63 antibody- or CD3 antibody- conjugated microbeads to meet the detection limitation of traditional flow cytometry. The accuracy of the acquired data, such as the percentage and fluorescence intensity of PD-L1-positive sEVs, was compromised by aggregates of sEVs on beads. Thus, the diagnostic and predictive potential of sEV PD-L1 in HNSCC should be further investigated and confirmed by single-particle analysis using nanoscale flow cytometer with higher resolution.

### Gastric cancer

Gastric cancer (GC) ranks fifth for cancer incidence and third for cancer-related death worldwide^34^. In human GC cell lines, Fan et al. found that the secreted level of sEV PD-L1 was associated with the expression level of PD-L1 in donor cells^29^. They also demonstrated that higher baseline level of circulating sEV PD-L1 was associated with lower overall survival (OS) in 31 metastatic GC patients receiving chemotherapy. The level of sEV PD-L1 was an independent prognostic factor in GC patients, while sPD-L1 could not predict the prognosis of GC. Additionally, the level of circulating sEV PD-L1 negatively correlated with the counts of CD4 and CD8 T cells or the level of granzyme B. These results suggest that the level of circulating sEV PD-L1 could serve as a promising biomarker for predicting the prognosis and immunosuppression status of metastatic GC. Recently, Zhang et al. reported that 5-fluorouracil (5-FU) increased sEV PD-L1 in patients with stage III-IV GC. Moreover, the level of circulating sEV PD-L1 was increased more significantly in clinical non-responders than responders^27^. However, the role of sEV PD-L1 in monitoring anti-PD-1 immunotherapy response in GC patients still needs further investigation.

### Pancreatic cancer

Due to that diagnosis is often made in advanced tumor stages, pancreatic cancer is counted among cancer diseases with the highest mortality with 5-year survival around 5%^39^. It is urgent to find the biomarkers for early diagnosis of pancreatic cancer. Lux et al. analyzed the level of sEV PD-L1 in 55 patients with pancreatic cancer, 26 patients with chronic pancreatitis (CP), and 10 patients with benign serous cyst adenoma of the pancreas^20^. However, no significant difference in level of sEV PD-L1 was found between pancreatic cancer and CP patients. The median postoperative survival time of patients with sEV PD-L1-positive pancreatic cancer (17.2 months) was significantly longer than that of sEV PD-L1-negative patients (7.84 months). These results indicate that the level of sEV PD-L1, which can serve as a negative prognostic factor, is not a good diagnostic biomarker for pancreatic cancer. It was reported that pancreatic cancer is one of the most stroma-rich cancer, which consists of a large amount of infiltrated immune cells that negatively regulate anti-tumor immunity^40^. A very recent study has shown that both tumor cells and immune cells secret PD-L1-positive sEVs into circulation, while only PD-L1-positive sEVs from tumor cells and CD8 T cells predict clinical outcome of treatment^41^, suggesting that the heterogeneity in cellular origin would significantly affect the accuracy of sEV PD-L1-based liquid biopsy. Thus, cell markers, especially tumor cell markers, should be introduced into the analysis of sEV PD-L1. Of note, glypican-1 (GPC1) has been previously reported to be enriched on pancreatic cancer-derived sEVs, being capable of detecting pancreatic cancer at early stages^42^. It can be therefore speculated that the diagnostic specificity of sEV PD-L1 in pancreatic cancer would be potentially improved by combining tumor cell markers (e.g., GPC-1) or other molecules that co-expressed on PD-L1-positive sEVs.

### Glioma

Glioma is the most common primary intracranial tumor, representing over 70% of malignant brain tumors^43^. Glioma has three subtypes, proneural (P), classical and mesenchymal (M). It has long been recognized as an immunosuppressive cancer with high expression level of PD-L1, especially in M glioma^44,45^. Garcia et al. revealed that PD-L1 was highly present in circulating sEVs from both grade 4 glioblastoma patients and normal donors, and the difference was not significant^18^. They claimed that sEV PD-L1 is not a reliable predictor in the diagnosis of glioblastoma as it is ubiquitous in normal donors. However, Muller et al. demonstrated that the level of PD-L1 mRNA in sEVs was useful in predicting glioma patients’ response to vaccination therapy^46^. Recently, Ricklefs et al. revealed that PD-L1 protein was present on the surface of some, but not of all, glioblastoma-derived sEVs. There was no significant difference in the level of sEV PD-L1 protein between glioblastoma patients and healthy donors^17^. The level of PD-L1 in cancer tissues determined by IHC assay (clone of the used anti-PD-L1 antibody: E1L3N) was positively correlated with the level of circulating sEV PD-L1 DNA in glioblastoma patients. As expected, the level of PD-L1 DNA in sEVs was positively correlated with the glioblastoma tumor volume (up to 60 mm^3^) determined by magnetic resonance (MR) imaging^17^. These results indicated that sEV PD-L1 DNA may serve as a reliable biomarker in early diagnose of glioblastoma.

### Breast cancer and other cancers

Among females, breast cancer is the most diagnosed cancer and the leading cause of cancer death^34^. Yang et al. recently evaluated the biological functions of sEV PD-L1 in breast cancer^3^. They found that the higher co-localization level of CD63 and PD-L1 was associated with the higher disease stage. The associations between the level of serum sEV PD-L1 and the clinicopathologic features and the clinical outcomes of oncotherapy still need further investigation in breast cancer patients in the future. Huang et al. recently examined the level of circulating sEV PD-L1 in 34 cancer patients (15 urothelial carcinoma, 11 gastric adenocarcinoma, 6 prostate adenocarcinoma, 1 ovarian sarcoma, 1 small cell lung cancer) and 22 healthy volunteers^23^. They demonstrated that the level of circulating sEV PD-L1 can effectively distinguish cancer patients from healthy donors. Moreover, the level of sEV PD-L1 was positively correlated with cancer stage and metastasis, indicating that sEV PD-L1 was a reliable predictive biomarker for disease progression in malignant cancers.

## The role of sEV PD-L1 in cancer immunotherapy

A lot of cancer patients failed to respond to checkpoint blockade therapy. It was supposed that patients with positive expression of PD-L1 in tumor tissue would benefit from PD-1/PD-L1 blockades. However, according to the results of several clinical trials, both PD-L1-positive and -negative cancer patients could benefit from anti-PD-1 blockade immunotherapy^47–49^. This is probably because the heterogeneity and dynamic changes of PD-L1 expression in tumor. Therefore, there is an urgent need to validate a biomarker allowing selection of the responders who might benefit from checkpoint blockade therapies. Recently, developing sEV PD-L1 as a blood-based biomarker has become an attractive option.

### sEV PD-L1 as a predictor for immunotherapy in melanoma

Melanoma is the most aggressive and deadly skin cancer that typically associated with ultraviolet exposure. The treatment and clinical outcome of metastatic melanoma has changed substantially over the past decade because of immunotherapy, especially the immune checkpoint inhibitor-based immunotherapy^50^. A number of reports, including our previous study in 44 patients with stage III to IV melanoma treated with pembrolizumab^1^, have shown that high levels of circulating sEV PD-L1 before the treatment were associated with poorer clinical outcomes of immunotherapy. However, based on the reported data including ours^1,4,41^, some melanoma patients with high level of sEV PD-L1 also showed good therapeutic response whereas some patients with low level of sEV PD-L1 did not respond to the therapy, suggesting that the pre-treatment level of sEV PD-L1 alone is imperfect to predict the patient response. In the meantime, available results have suggested that both the mRNA and protein levels of sEV PD-L1 may change during the therapy and the evolution dynamics are closely associated with the patient response. It was revealed that, in patients receiving anti-PD-1 therapy, the level of PD-L1 mRNA in sEVs was decreased in patients with complete response/partial response (CR/PR), while it was increased in patients with progression of disease (PD), when compared with the baseline levels^51^. No significant changes were observed in patients with stable disease (SD). Consistently, we previously found that the protein level of circulating sEV PD-L1 was positively correlated with the level of circulating IFN-γ and overall tumor burden. Early after immunotherapy was started (at 3–6 week of treatment), PD-1 blockade increased IFN-γ production by PD-1^+^CD8^+^ T cells. The increased IFN-γ in turn induced the secretion of sEV PD-L1 in melanoma cells, resulting the increased level of sEV PD-L1 both in responders and non-responders. However, the magnitude of increase in sEV PD-L1, which reflected T cell reinvigoration and adaptive immune activation, was significantly higher in responders^1^. Moreover, this increased sEV PD-L1 would not be expected to inactivate CD8 T cells as the interaction between PD-L1 and PD-1 was blocked by anti-PD-1 therapy. The results of ROC analysis showed that a larger fold change of sEV PD-L1 level at week 3–6 could serve as a reliable biomarker to stratify patients by clinical response to anti-PD-1 therapy. According to another study, which evaluated the variations of sEV PD-L1 protein level before and months after anti-PD-1 therapy (median interval was 4.5 months) in 100 melanoma patients, a decrease in sEV PD-L1 after treatment is associated with response to treatment, while a high increase in sEV PD-L1 is associated with tumor progression^4^. These two studies together suggest that the protein level of sEV PD-L1 changes during and after immunotherapy, and the tendency and amplitude of this change can be used for distinguishing responders from non-responders. In particular, an increase in the level of sEV PD-L1 could be observed early during the treatment and late after the treatment, respectively. However, the increase in sEV PD-L1 at different stages may represent distinct therapy response and disease status. A larger increase in the level of sEV PD-L1 at 3–6 weeks following the initial treatment, reflecting a successful anti-tumor immunity elicited by the treatment, was usually observed in responders. After several months of treatment, however, obvious tumor regression would occur in responders, leading to a decrease in the secretion level of sEV PD-L1. By contrast, the non-responders would experience significant tumor progression 4.5 months after treatment, which may result in an increase in the secretion level of sEV PD-L1. Therefore, the significantly increased level of sEV PD-L1 after months of treatment, probably associating with the increased tumor burden, was usually observed in non-responders. Although the dynamics of sEV PD-L1 during and after treatment could be utilized to improve the prediction performance, it takes months to monitor the change and predict the response, which is despairing for non-responders to seek other effective treatment. Therefore, strategies to increase the specificity and sensitivity of pre-treatment sEV PD-L1-based biomarker should be developed in the future, such as simultaneous detection of other co-expressing molecules and exploitation of new detection methods. Of interest, a very recent study revealed that sEV PD-L1 in melanoma patients were secreted by both tumor cells and immune cells, and only tumor cell- and CD8 T cell-derived PD-L1-positve sEVs were significantly higher in non-responders than in responders^41^. As the result, melanoma cell maker CD146 combined with PD-L1 on sEVs exhibited a stronger efficiency than PD-L1 alone for predicting immunotherapy response^41^.

### sEV PD-L1 as a predictor for immunotherapy in lung cancer

Currently, nearly all patients with metastatic NSCLC receive anti-PD-1/PD-L1 therapy in first-line setting except for the metastatic NSCLC harboring targetable oncogenes^52^. Re et al. revealed that the level of sEV PD-L1 changed in NSCLC patients receiving nivolumab and pembrolizumab^51^. Specifically, the number of PD-L1 mRNA copies in circulating sEVs significantly increased in subjects with disease progression and decreased in patients responding to treatment with nivolumab. While no significant changes were observed in patients with SD. To improve the prediction accuracy, a combination of multi-marker parameters has been developed recently. Zhang et al. investigated the level of sEV PD-L1 in a total of 44 patients with several types of advanced tumors, including 27 (61.4%) lung cancer, treated by anti-PD-1 therapy. They found that low baseline level of sEV PD-L1 and high level of CD28 could screen out the potential beneficiary patient cohort of anti-PD-1 therapy^53^. These findings suggest that the levels of sEV PD-L1 and CD28 could serve as the predictive biomarkers for clinical responses to anti-PD-1 treatment.

### sEV PD-L1 as a predictor for immunotherapy in HNSCC

Theodoraki et al. has investigated whether the level of tumor-derived or T cell-derived PD-L1-positive sEVs can predict clinical outcomes of HNSCC patients (*n* = 18) treated with a combination of cetuximab, ipilimumab, and radiation therapy. They found that patients with high level of CD3^−^PD-L1^+^ sEVs at baseline might benefit from immunotherapy. In patients with recurred disease, the level of CD3^−^PD-L1^+^ sEVs increased from the baseline level. A therapy-induced decrease in the level of CD3^−^PD-L1^+^ sEVs was only observed in patients with NED^54^. As aforementioned, PD-L1-positive sEVs in cancer patients may be secreted by both tumor and non-tumor cells. Moreover, the heterogeneity in cellular origin would significantly affect the accuracy of sEV PD-L1-based liquid biopsy. Therefore, other co-expressing molecules on PD-L1-positive sEVs, such as cell markers, should be simultaneously determined in the future to improve the prediction of immunotherapy in HNSCC.

## Current challenges and future perspectives of sEV PD-L1

### Selective purification of PD-L1-positive sEVs with natural properties

#### Surface marker-based purification of sEV PD-L1

Usually, PD-L1-positive sEVs were intermingled with PD-L1-negative sEVs in body fluids or cell culture supernatants. In order to elucidate their unique molecular profiles and biological behaviors, it is necessary to obtain the purified PD-L1-positive sEVs. Among the commonly used isolation methods, surface marker-based isolation, such as the immunoaffinity-based capture technique, is seemingly more appropriate for the selective purification of PD-L1-positive sEVs. Theoretically, the immunoaffinity-based capture strategy, which depends on the binding between PD-L1 and anti-PD-L1 antibody-conjugated magnetic beads, almost has no adverse effects on subsequent sEV cargo analysis. However, it would be difficult to release PD-L1-positive sEVs from the magnetic beads without affecting the integrity of sEVs. Undoubtedly, the beads, which are usually significantly larger and heavier than sEVs, will dramatically change the physical properties and biological function of sEVs. Moreover, even the beads were removed by breaking the connection between beads and antibodies, the PD-L1 protein on sEV surface would be still masked by the antibodies, thereby resulting in a different performance of PD-L1-positive sEVs in vitro and in vivo. For instance, the reported results have demonstrated that blocking sEV PD-L1 with anti-PD-L1 antibodies will inhibit their immunosuppressive effects^1,3,33^.

#### Nondestructive purification of PD-L1-positive sEVs

**Fig. 5:**
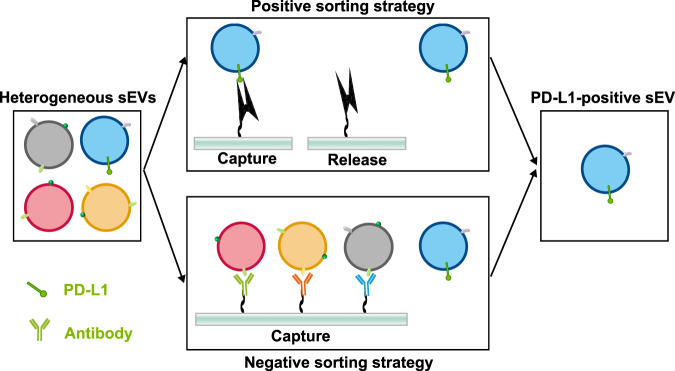
The nondestructive isolation strategies for selective enrichment of sEV PD-L1. Nondestructive isolation strategies for PD-L1-positive sEVs generally fall into two major categories, the ‘positive sorting strategy’ and ‘negative sorting strategy’.

To identify their fundamental characteristics and biological functions more accurately, the first and essential step is to separate PD-L1-positive sEVs from PD-L1-negative sEVs. However, as aforementioned, the currently existing isolation methods (e.g., ultracentrifugation, size-based isolation technique and immunoaffinity-based capture technique) are inapplicable to the selective separation of PD-L1-positive sEVs. Thus, strategies for selective purification of PD-L1-positive sEVs that could maximally preserve their natural properties and functions are highly needed. According to the isolation strategy, nondestructive isolation strategies generally fall into two major categories, the “positive sorting” and “negative sorting” (Fig. 5). In positive sorting strategy, PD-L1-positive sEVs are directly separated by isolation agents. While in negative sorting, PD-L1-positive sEVs were passively enriched by depleting the unwanted sEV subtypes. Aptamer, also known as chemical antibody, binds to their targets with high specificity and affinity. Moreover, aptamer can be easily removed from their targets by corresponding complementary sequences without disturbance to the natural characteristics of targets. Thus, the aptamer-based magnetic isolation strategy holds tremendous potential in the specific capture and nondestructive release of sEV PD-L1. In negative sorting strategy, the isolation agents were only binding with unwanted sEVs, preserving the natural characteristics of target sEVs. The subpopulations of PD-L1-positive sEVs with different surface markers can be further purified using negative sorting. Additionally, the negative sorting strategy can compensate for the limited quantity of aptamer, which was restrained by tedious screening process.

### The heterogeneity of PD-L1-positive sEVs

#### The heterogeneity of PD-L1-positive sEVs in cellular origin

**Fig. 6:**
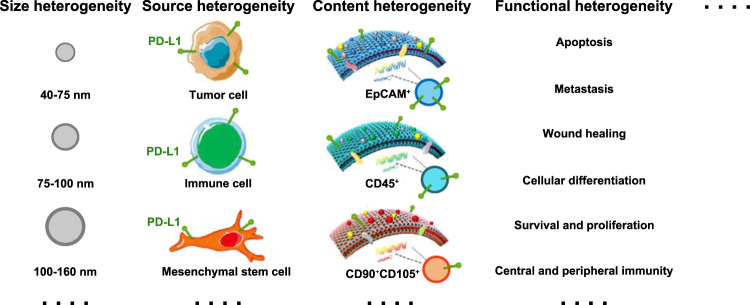
The heterogeneity of sEV PD-L1. The heterogeneity of sEV PD-L1 may be conceptualized based on their size, cell of origin, content, and functional on recipient cells.

In addition to selective purification, another challenge that would hinder our understanding of the fundamental characteristics and biological functions of PD-L1-positive sEVs is their heterogeneity. The heterogeneity of PD-L1-positive sEVs is generally referred to the differences in their cellular origin, size, content, and functional impact on recipient cells (Fig. 6). PD-L1 is not exclusively expressed on tumor cells. Non-tumor cells, such as T cells, natural killer (NK) cells and dendritic cells (DCs), also carry PD-L1^55,56^. Apparently, sEV PD-L1 from those PD-L1-bearing non-tumor cells also contribute to the pool of total sEV PD-L1^38,41^, suggesting the cellular origin heterogeneity of sEV PD-L1. However, the biological functions of those non-tumor cell-derived PD-L1-positive sEVs remain unclear. Additionally, in order to improve the accuracy of prediction, whether the non-tumor cell-derived sEV PD-L1 need to be excluded when we select the patients who may benefit from anti-PD-1/PD-L1 therapies is still need to be revealed.

#### The heterogeneity of PD-L1-positive sEVs in size distribution

The size distribution of sEVs was identified as smaller than 200 nm. Recently, improved analytical tool for isolating sEVs has revealed that sEVs contain subpopulations defined by a distinct size range (larger sEV vesicles and small sEV vesicles)^57^. Studies have revealed that some proteins prefer to enrich on the sEVs with certain size^58^. For instance, CD63 were mainly presented in the sEVs smaller than 50 nm, while MHC-II molecules were mainly enriched in sEVs with diameters larger than 100 nm. Up to now, the size distribution of PD-L1-positive sEVs remain unclear. The characteristics of PD-L1-positive sEVs with different size need to be investigated in the future.

#### The heterogeneity of PD-L1-positive sEVs in content and function

Due to the heterogeneity of the PD-L1-positive sEVs in origin and size, they may have some crucial biological differences in the molecular composition, denoted as the content heterogeneity. Moreover, it has been traditionally expected that sEVs derived from the same cells or cell lines contain similar protein, nucleic acid, and lipid composition. However, recent studies have revealed that the molecular composition of sEVs is not only cell-type dependent but would be different even when the sEVs were originated from the same donor cells^59^. This suggests that a comprehensive understanding of their composition at individual and population levels is required.

The heterogeneous PD-L1-positive sEVs horizontally transfer their cargo to recipient cells and change the behaviors of recipient cells. The content heterogeneity is considered as the theoretical basis of the different effects of sEV PD-L1 on recipient cells, named as the functional heterogeneity of sEV PD-L1. The functional heterogeneity of sEV PD-L1 refers to the distinct behaviors of same receipt cells stimulated by heterogeneous of sEVs in size, cellular origin, and molecular. It also refers to the distinct response of different receipt cells to the same sEVs.

#### Single-particle assay for PD-L1-positive sEVs

The heterogeneity cause variation of the molecular profiles of individual PD-L1-positive sEVs. Due to their heterogeneity, bulk analysis of total PD-L1-positive sEVs is insufficient to accurately identify the disease state. Thus, the single-particle assay that distinguishes their cellular origin, size, content, and function on recipient cells is the future direction of the detection of sEV PD-L1. This refined detection strategy may answer the question why some PD-L1-positive patients could not benefit from PD-L1/PD-1 blockade immunotherapy. In this regard, single-particle assay for PD-L1-positive sEVs is of vital significance for the precise diagnosis and analysis of diseases. Therefore, we hold that it is important to develop a clinical available detection method for single-particle analysis of PD-L1-positive sEVs in the future.

### sEV PD-L1 as diagnostic and predictive biomarker

#### sEV PD-L1 subpopulations as more accurate biomarkers

As mentioned earlier, an exponentially increasing number of studies have demonstrated that sEV PD-L1 could be the tumor diagnostic markers. Moreover, monitoring of circulating sEV PD-L1 may serve as an indicator to stratify clinical responders from non-responders in immunotherapy. However, currently, the applicability and accuracy of sEV PD-L1-based biomarker are imperfect. More dramatically, the predicting outcomes varied with different tumor types, detection methods, the timing of specimen collection or even different research groups. A key reason for the disparity is the heterogeneity of sEV PD-L1, which refers to their distinct cellular origin, size, and molecular profiles. In order to improve the accuracy of sEV PD-L1-based liquid biopsy, which subpopulation of sEV PD-L1 should been employed remains unclear. Nonetheless, we still hold that the subtype detection of sEV PD-L1, which will reveal more information about sEV PD-L1, will bring a more accurate diagnostic and predictive biomarker.

#### The specificity and sensitivity of sEV PD-L1-based biomarker

Although the level of sEV PD-L1 has been suggested as a potential diagnostic marker in different tumor types, the predictive sensitivity and specificity of the pre-treatment level of sEV PD-L1 in immunotherapy has been controversial. Several studies have confirmed that the level of sEV PD-L1 changes during and after immunotherapy, and the tendency and amplitude of this change can be superior to the pre-treatment level of sEV PD-L1 for distinguishing responders from the non-responders. Using ROC curve analysis, we previously reported that the fold change of sEV PD-L1 during early stage (at 3–6 week of treatment) of anti-PD-1 therapy was a biomarker to distinguish responders from non-responders with a sensitivity 80.00% and specificity 89.47%^1^. Cordonnier et al. also reported that the variations of sEV PD-L1 before and months after anti-PD-1 therapy (median interval was 4.5 months) showed good discrimination between responders and non-responders, showing an 83% sensitivity, a 70% specificity, a 91% positive predictive value and a 54% negative predictive value for disease progression^4^. Although the dynamics of sEV PD-L1 during and after treatment could be utilized to improve the prediction performance, it takes months to monitor the change and predict the response, which is despairing for non-responders to seek other effective treatment. Therefore, strategies to increase the specificity and sensitivity of pre-treatment sEV PD-L1-based biomarker should be developed in the future, such as simultaneous detection of other co-expressing molecules and exploitation of new detection methods.

#### Standardization of sEV PD-L1-based liquid biopsy

The reported level of sEV PD-L1 and its predictive accuracy varied with tumor types. The detection methods or even the operators may greatly hinder the popularization and promotion of sEV PD-L1-based liquid biopsy. Thus, developing and implementing a standard methodology and normal range for the detection of sEV PD-L1 in different tumor types is a matter of great urgency to unlock their potential in liquid biopsy.

### Application of sEV PD-L1 in therapies: eliminate them or exploit them

#### Specific inhibition of the biogenesis and secretion of sEV PD-L1

Due to the immunosuppressive and tumor-promoting effects, inhibition of sEV PD-L1 would serve as an effective strategy to disrupt the growth of various tumors in the future. The combination between sEV PD-L1 inhibition and PD-L1/PD-1 blockade may be a promising strategy to effectively suppress tumor growth in the clinic, yielding new ways to overcome the resistance to anti-PD-1/PD-L1 therapy^60^. The most promising strategy to counteract their adverse effects is blocking the generation of sEV PD-L1. Apparently, elucidation of the precise biogenesis and secretion mechanism of sEV PD-L1 is an essential prerequisite for the strategies to inhibit their generation. However, the molecular mechanisms have not been fully understood until now. Recent studies, including our work, reported several molecules, including Rab27, Alix, Hrs, and nSMase2, participated in the generation, sorting, transportation, and secretion of sEV PD-L1. Thus, targeted inhibitors of Rab27, Alix, Hrs and nSMase2 should be fully exploited in the future. Since these reported regulatory molecules are also involved in the biogenesis and secretion of sEVs from non-normal cells, inhibitors should be specifically delivered to tumor cells. Moreover, the combination of multiple inhibitors, which play the leading role during different stages of sEV PD-L1 generation, may lead to enhanced inhibitory effect on sEV PD-L1. Meanwhile, new key molecular or signal pathway involved in the generation of sEV PD-L1 should be also further exploited.

#### Selective clearance of the existing PD-L1-postitive sEVs

Until now, there is no convincing evidence that any drug can selectively inhibit the generation of sEV PD-L1. Even if targeted inhibitors were eventually developed in the future, it would be difficult to entirely suppress the generation of sEV PD-L1. Moreover, sEV PD-L1, existing in the extracellular space, and its biological effects might last for a long period of time before they are cleared by organism. Therefore, clearance of sEV PD-L1, which already exists in the extracellular space, should also be developed as alternatives, or complements to blocking the generation of sEV PD-L1. We hold that blocking the biogenesis of sEV PD-L1 is shutting off the water inlet, which prevents the unwanted impact of sEV PD-L1 from source, and clearance of PD-L1-positive sEVs is turning the drainpipe of sEV PD-L1 pool. One accessible method is the therapeutic plasma exchange (TPE). Orme et al.^61^ recently reported that TPE can clear total circulating sEVs including PD-L1-positive sEVs. Like dialysis, blood is passed through an apheresis machine that separate plasma from PD-L1-positive sEVs. In this strategy, normal cell-derived sEVs, which may responsible for normal physiological functions, were also passively cleared. An alternative method to eliminate circulating sEV PD-L1 is to promote the selective clearance of PD-L1-positive sEVs by shortening their lifetime in the future. However, the lifetime and mechanism of the clearance of PD-L1-positive sEVs from circulation remain uncertain at presents.

#### Usage of sEV PD-L1 in suppressing the hyperactive immune system

Until now, studies about sEV PD-L1 were mainly focused on tumor, especially on their immunosuppressive effects in tumor development. The role of sEV PD-L1 beyond tumor are far from being fully understood. Autoimmune diseases are diseases that occur in which the hyperactive immune system attacks healthy organs, tissues, and cells in the body. Immunosuppressive sEV PD-L1 could be developed as immunosuppressant to suppress the hyperactive immune system of patients with autoimmune diseases. Among them, stem cell-derived sEVs, which carry PD-L1 and serve as cell-free therapies, have the capacity to counteract with autoimmune diseases. Additionally, the immunosuppressive sEV PD-L1 also has potential in the treatment of other pathological conditions with overactivated immune response (e.g., inflammatory diseases and wound healing). Su et al. revealed that tumor cell-derived PD-L1-positive sEVs speed up wound healing by inhibiting the function of T cells^62^. Unquestionably, the effects of sEV PD-L1 beyond tumor, which should be consistently explored in the future, are currently being uncovered. On the other hand, the biological functions of PD-L1-positive sEVs from non-tumor cells, especially the immunocytes, remain entirely unclear. Whether they share similar immunosuppressive effects with that from tumor cells should be further explored.

## Conclusion

In summary, our present review systematically reviewed the current research status of sEV PD-L1 and prospected several potential challenges and future directions based on our ongoing studies and pioneering understandings. The potential challenges and focuses of sEV PD-L1 research in the following aspects: (1) selective purification of PD-L1-positive sEVs with natural properties and functions; (2) heterogeneity of PD-L1-positive sEVs; (3) standardization of sEV PD-L1-based liquid biopsy; (4) novel cancer therapies based on sEV PD-L1. By highlighting these important knowns and unknowns, we hope to light the way forward for the research field of sEV PD-L1 and to avoid unnecessary blindness and detours.

## Data Availability

No datasets were generated or analyzed during this study.
